# Supplementary material for: Platelets regulate neural and oligodendroglial progenitors when infiltrating the brain parenchyma
Source: Commun Biol. 2025 Nov 24;8:1640. doi: 10.1038/s42003-025-09028-1 (PMC12644571; doi:10.1038/s42003-025-09028-1)
Supplement: Supplementary file 2 — Supplementary Information [file 42003_2025_9028_MOESM2_ESM.pdf]

Supplementary Material

Title:

Platelets regulate neural and oligodendroglial progenitors when infiltrating the brain parenchyma

Authors:

Christina Dimitriou[1*], Maria Anesti[1*], Stefanos Kaplanis[2], Theodora Mourtzi[1], Aggeliki Dimopoulou[1], Dimitrios Dimitrakopoulos[1], Filippos Katsaitis[1], Maria Nousia[1], Konstantina Mastori[1], Kyriaki Karassavidou[1], Efthimia Aleiferi[1], Dimitrios Lagogiannis[1], Esra Tahir[8], Amber R Philp[3], Adamantia Kouvela[4], Constantinos Stathopoulos[4], Domna Karagogeos[2], Robin JM Franklin[5], Cedric Ghevaert[6], Francisco J Rivera[3,7], Ilias Kazanis[1,5,8]

*: equally contributing

Corresponding author:

Dr Ilias Kazanis, School of Life Sciences, University of Westminster, London, UK. i.kazanis@westminster.ac.uk

Affiliations:

1. Laboratory of Developmental Biology, Department of Biology, University of Patras, Patras, Greece

2. Department of Basic Science, University of Crete Medical School and Institute of Molecular Biology and Biotechnology, Foundation for Research and Technology Hellas, Heraklion, Greece

3. Laboratory of Stem Cells and Neuroregeneration, Institute of Anatomy, Histology and Pathology, Faculty of Medicine, Universidad Austral de Chile, Valdivia, Chile & Center for Interdisciplinary Studies on the Nervous System (CISNe), Universidad Austral de Chile, Valdivia, Chile

4. Department of Biochemistry, School of Medicine, University of Patras, 26504 Patras, Greece

5. Cambridge Stem Cell Institute & Department of Clinical Neurosciences, University of Cambridge, Cambridge CB2 0AW, UK

6. Cambridge Stem Cell Institute & Department of Haematology and NHS Blood and Transplant, University of Cambridge, Cambridge, UK

7. Translational Regenerative Neurobiology Group (TReN), Molecular and Integrative Biosciences Research Program (MIBS), Faculty of Biological and Environmental Sciences, University of Helsinki, Finland

8. School of Life Sciences, University of Westminster, London, UK

Supplementary Material

Supplementary Figures and Legend to Supplementary Video1

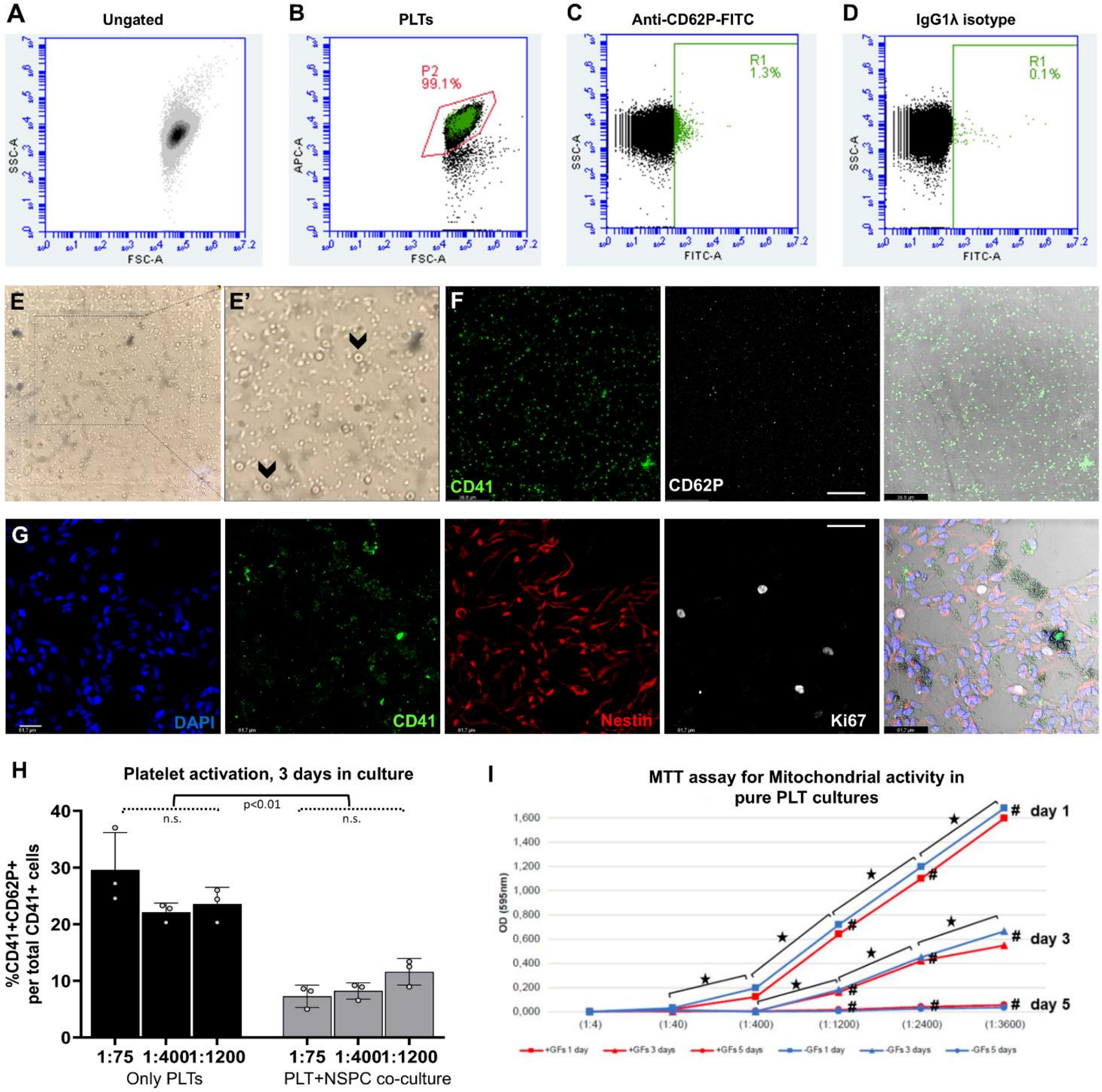

Supplementary Material

Supplemental Figure 1. Isolation of washed platelets

[A- D] Scatterplot matrices of washed, isolated, platelets (PLTs) after FACSorting. Side and forward scatter plots are shown in (A) and almost 99% of the events were immunopositive for CD41 (B). Out of the CD41+ events, only 1.3% were co-expressing the activated platelet marker CD62P (C). The pool of activated platelets is not visible after immunostaining for an isotype control (D). [E] Isolated and washed platelets are shown in brightfield and in higher magnification in E'. Note the presence of few erythrocytes, that are out of focus, indicated by arrows. [F] Isolated and washed platelets were plated on glass coverslips and immunostained for CD41 and for CD62P. [G] Microphotographs of NSPCs (immunopositive for nestin, in red) co-cultured with platelets (1:1200 ratio, immunopositive for CD41, in green). Proliferating cells are detected by the expression of Ki67 (in white). On the right of the panel fluorescence is merged with brightfield. [H] Graph showing the fraction of CD41+ cells that co-express CD62P when platelets were plated alone (dark grey bars), or co-cultured with NSPCs (light grey bars), in the respective densities (presented as NSPC:platelet ratios). [I] Graph showing the optical density using the MTT assay, to measure mitochondrial activity in pure platelet cultures (in densities respective to those used in NSPC:platelet co-cultures), in the presence/ absence of growth factors (GF), after 1, 3 and 5 days. When platelets become activated, they also become exhausted and their mitochondrial activity is reduced. Note that in low densities (up to 1:40) full exhaustion / activation is observed even in day 1. Note also, that the higher the densities of platelets the lower the levels of activation on days 1 and three. Finally, note that after 5 days in culture almost all platelets have been activated and their mitochondria have been exhausted.

[scale bars: 35µm in F; 51µm in G. The bars show mean values and error bars the SEMs. 2-way ANOVAs were performed, followed by Tukey post-hoc analyses. In (G) the significant difference of the effect of the "culture conditions" (presence of NSPCs) is shown with the horizontal bracket and the respective p value. No significant effect of "time" was found. In (H) the stars indicate significant differences between different platelet densities (shown by the horizontal bracket lines), within the same time-point. The hash marks indicate significant differences between similar platelet densities, but on different time-points. "n.s."= non significant]

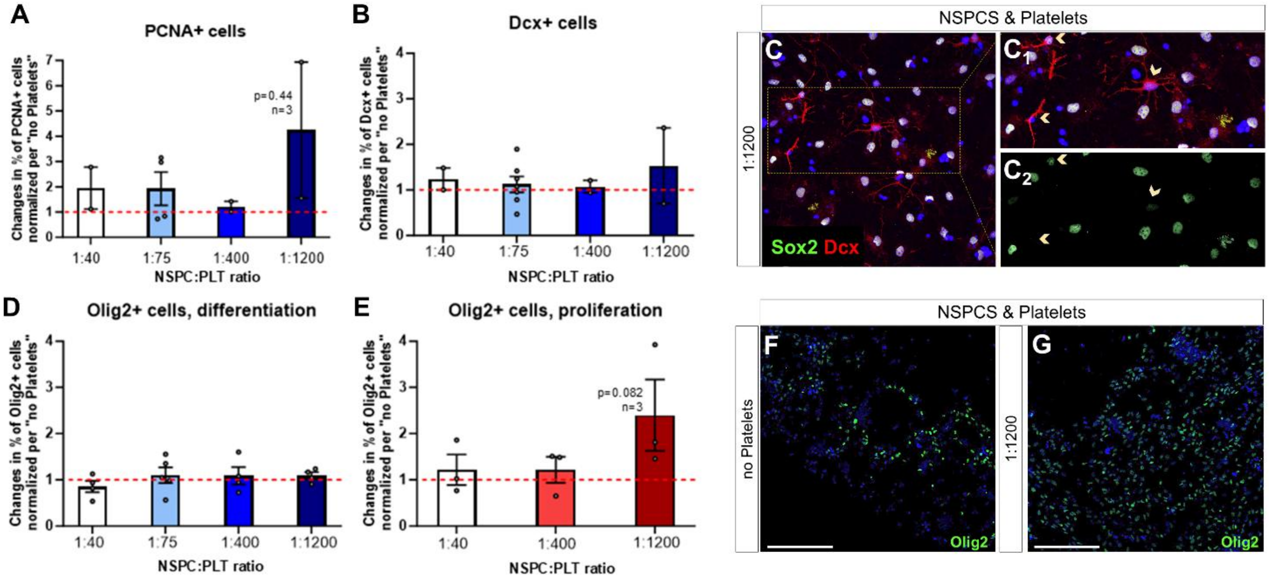

Supplemental Figure 2. Co-culture of platelets with NSPCs

Graphs showing the changes in the percentage of PCNA+ (in A), Dcx+ (in B) and Olig2+ (in D, E) cells, after co-culturing NSPC and platelets (PLTs) at different ratios in differentiation (in A, B, D) or proliferation (in E) conditions. The scatter plot bars show mean values and error bars the SEMs. The value of each NSPC biological sample (formed by 1 to 3 technical replicates) is depicted with a circle and is normalized per the “no-platelets” culture of the same sample, which is at “1.0” and is indicated by the red, dotted line. [C] Microphotograph of a NSPC:PLT co-culture in differentiating conditions and at a ratio of 1:1200. SOX2 immunopositive nuclei are shown in green and Dcx immunopoisitive immature neurons are shown in red (DAPI+ nuclei in blue). The arrowheads indicate examples of Dcx+ cells. [F, G] Microphotographs of NSPCs cultured on their own (in F), or co-cultured with PLTs, in proliferation conditions. Cells were immunostained for Olig2 (in green).

[Scale bars: 155µm. Statistical analyses were performed with 1-way ANOVA, followed by the Tukey post-hoc analysis. P-values are shown in (A) and (E) to clarify the absence of statistical significance.]

Supplementary Material

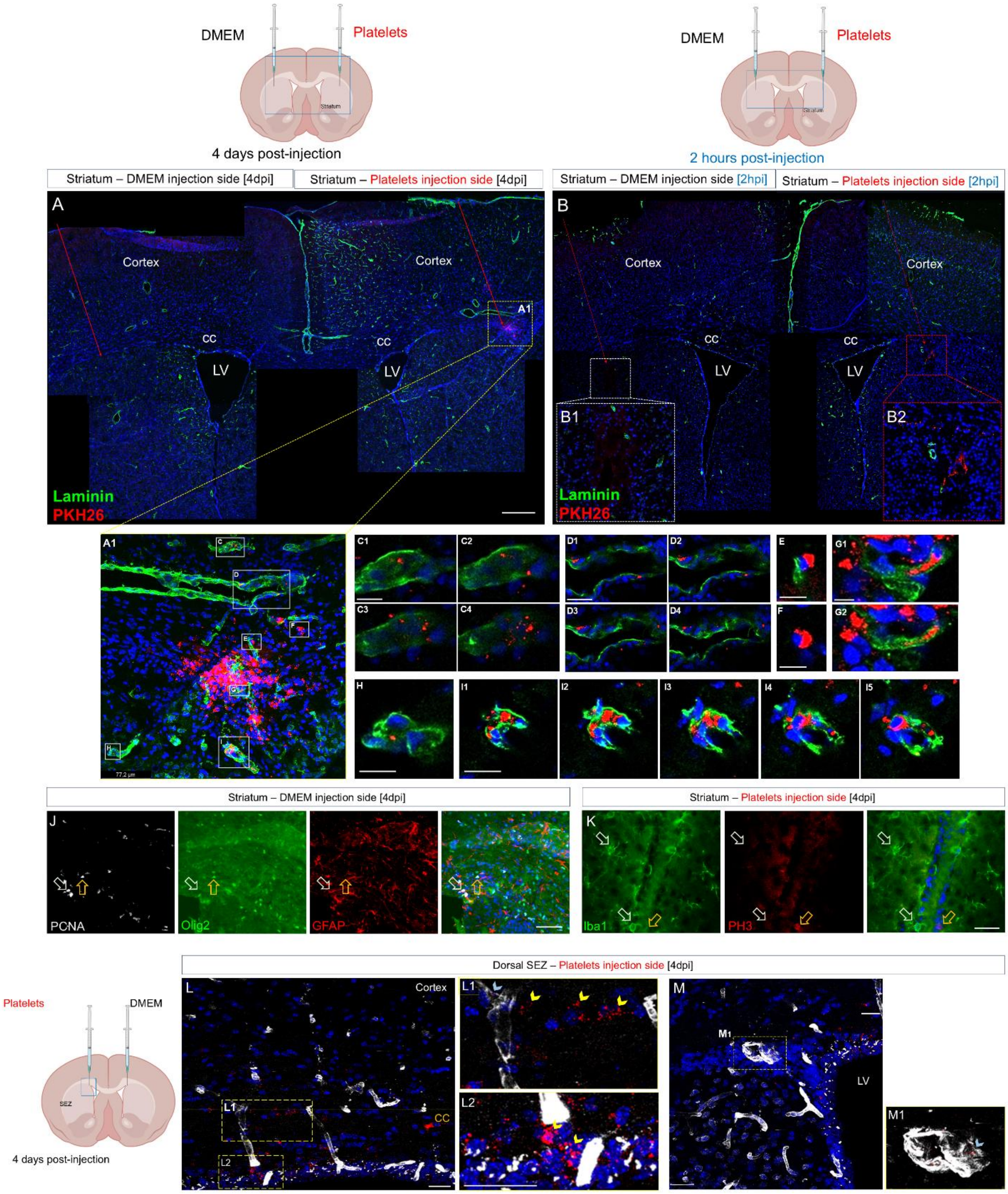

Supplemental Figure 3. Intracerebral injection of platelets

Supplementary Material

[A- B] Collages of microphotographs of mouse brain sections at 4 days (A) and 2h (B) after the intrastriatal injection of platelets labelled with PKH-26 (with red fluorescence) in one hemisphere and of DMEM in the contralateral side, after immunostaining for laminin (in green). The area of platelet injection is shown in higher magnification in (A1, B2) and the area of DMEM injection in (B1). Note the presence of red-dyed platelets in the parenchyma. Higher magnification details are shown in (A2) and in (C- I), with some examples of consecutive optical sections shown with numbers. Note the cases of platelets having entered within the vasculature. [J] Microphotograph taken at the site of injection of DMEM in the same, striatal, area, to show representative immunostaining for PCNA (in white), Olig2 (in green) and GFAP (in red). The white arrow indicates a mitotic, GFAP+ astrocyte and the yellow arrow a mitotic, Olig2+ OPC. [K] Microphotograph taken at the site of injection of PKH26-labelled platelets in the striatum, to show representative immunostaining for Iba1 (in green) and PH3 (in red). The white arrow indicates a microglial cell and the yellow arrow a mitotic nucleus. [L- M] Immunostainings (with laminin in white and PKH26 in red) from the area of platelet injection at the dorsal part of the SEZ, including the cortex and the corpus callosum (cc). Higher magnifications are shown in (L1, L2, M1). Examples of PKH-26-labelled platelets in the brain parenchyma are indicated with yellow arrowheads and examples of platelets within local blood vessels are indicated with magenta arrowheads.

[scale bars: 100μm in A, B; 5μm in C- I; 30μm in J- K; 50μm in L- M. LV: lateral ventricle]

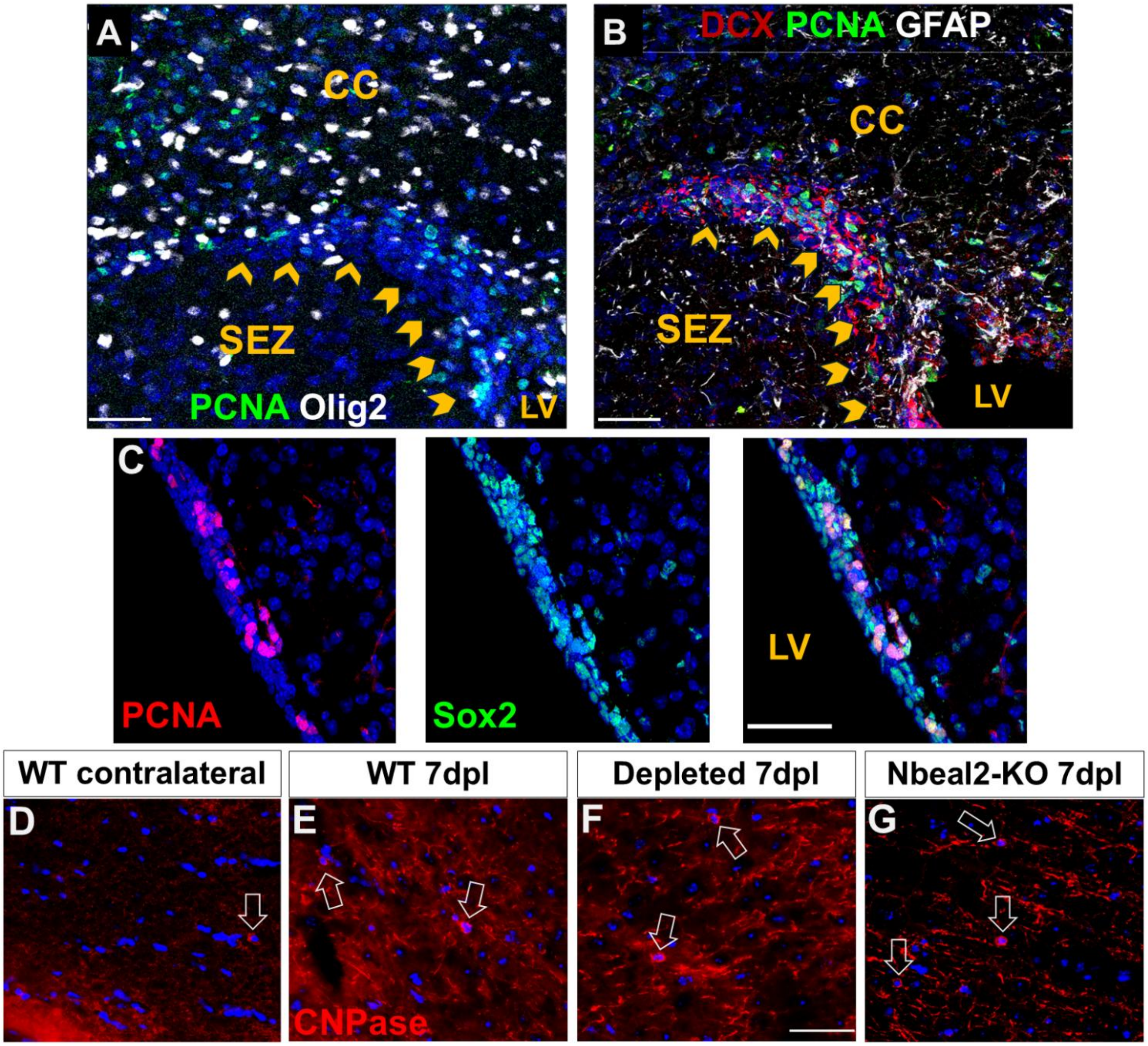
A
CC
SEZ
PCNA Olig2
LV
B
DCX PCNA GFAP
CC
SEZ
LV
C
PCNA
Sox2
LV
WT contralateral
WT 7dpl
Depleted 7dpl
Nbeal2-KO 7dpl
D
E
CNPase
F
G

Supplemental Figure 4

[A- C] Representative microphotographs of the Corpus Callosum (CC) and of the dorsal SEZ (in A, B; with its position indicated by the yellow arrowheads) and of the SEZ (C, adjacent to the Lateral Ventricle (LV)), in mouse brain sections from the control hemisphere of WT mice, after immunostaining for PCNA (in green, in A, B; red, in C), Olig2 (in white in A), Dcx (in red in B), GFAP (in white in B) and Sox2 (in green in C). (E- G) Microphotographs of the CC in mouse brain sections from the control, uninjured, and the demyelinated hemisphere of WT, Nbeal2 KO mice and of platelet-depleted mice at 7dpl, immunostained for CNPase. Examples of CNPase+ cells are shown with white arrows.

[scale bars: 50µm]

Supplementary Material

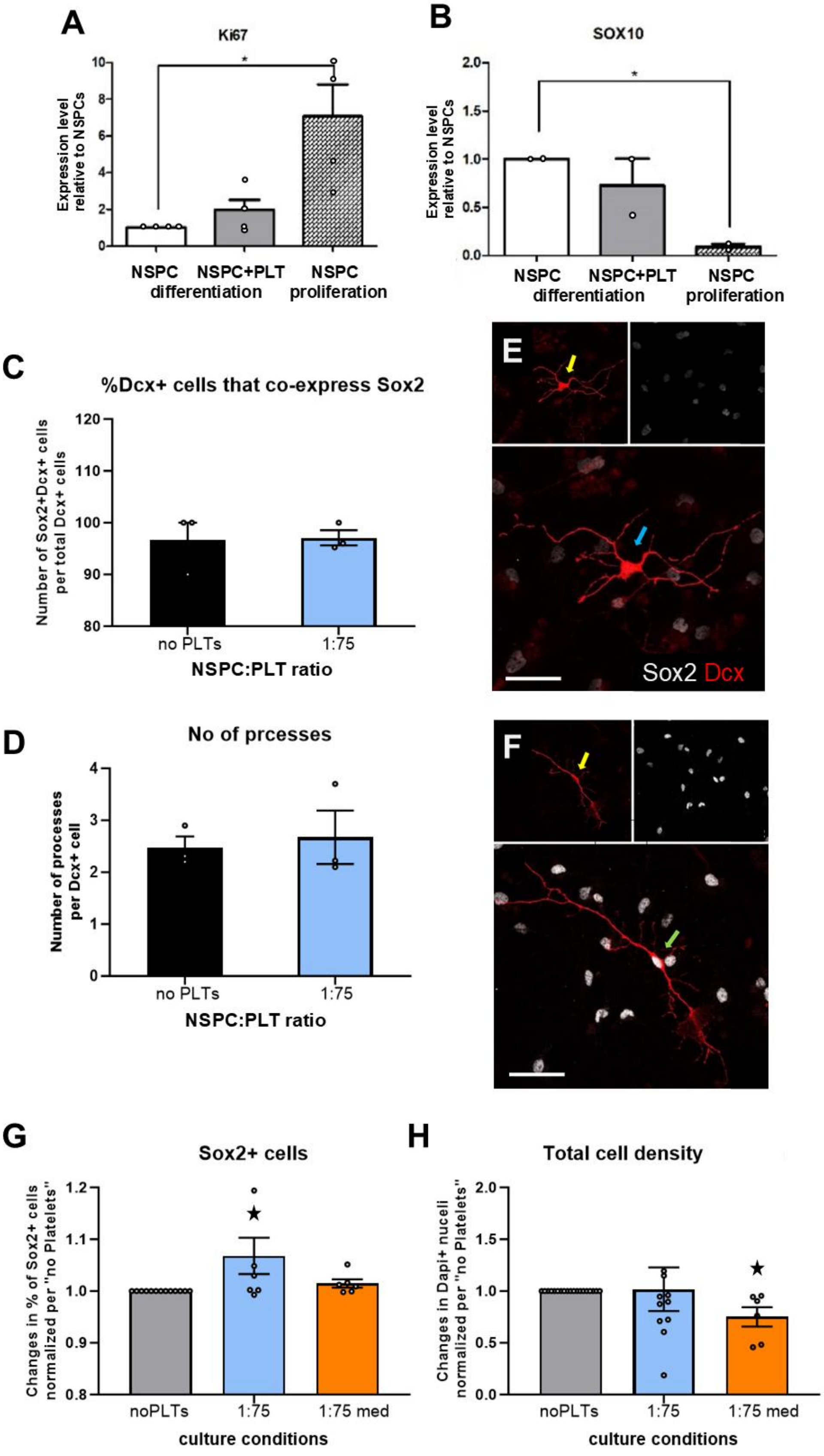

Supplemental Figure 5. Assessment of NSPC/platelet co-cultures

[A, B] Graphs showing the relative levels of the mRNA of Ki67 (A) and of Sox10 (B) in pure NSPC cultures in differentiation (Diff, white bars) or proliferation (Prol, textured bars) conditions, as well as in NSPC/platelet (PLT) co-cultures in differentiation conditions and at a respective ratio of 1:75. Note that in the co-cultures, the expression of Ki67 and of Sox10 in NSPCs is not different from the expression in the other two conditions, that differ significantly in between them. [C- F] Graphs showing the percentage of Dcx+ cells that co-express Sox2 (C) and the number of cell processes per Dcx+ cell (D), in NSPC cells cultured on their own in differentiation conditions (no-PLTs, in black) and in NSPCs co-cultured with PLTs, at a 1:75 ratio, in differentiation conditions (blue bars). Examples of Dcx+ (in red) and Sox2+ (in white) cells are shown in the microphotographs in (E) and (F). Dcx+ cell bodies are indicated with yellow arrows, with a Sox2- neuroblast indicated by a blue arrow (in E) and a Sox2+ neuroblast indicated by the green arrow (in F). [G, H] Graphs showing the percentage of Sox2+ cells (G) and the total cell density (H), in NSPC cells cultured on their own in differentiation conditions (no-PLTs, in grey), in NSPCs co-cultured with PLTs, at a 1:75 ratio, in differentiation conditions (blue bars) and in NSPCs cultured on their own in differentiation medium conditioned from a NSPC/PLTs co-culture, of a 1:75 ratio (yellow bars).

[scale bars: 25μm. Bars show the mean values and error bars the SEMs. 1-way ANOVAs were performed, followed by Tukey post-hoc analyses. Statistical differences ($p<0.05$) between two experimental conditions are shown with brackets and a star in (A, B). In (G, H) the stars indicate statistical differences ($p<0.05$) compared to both other conditions]

Supplemental Video 1

3D reconstruction of a blood vessel, identified by the expression of laminin (in red) with platelets identified by expression of CD41 (in green), within the SEZ niche of a wild-type mouse after the induction of a demyelinating lesion in the adjacent corpus callosum. Cell nuclei are counterstained with DAPI (in blue).

Supplementary Material

Materials and Methods

Isolation of platelets and assessment of mitochondrial activity

Platelets were isolated from the inferior vena cava[1]. Briefly, mice were given an i.p. overdose of ketamine (200µg per g of body weight). The thorax was exposed and approximately 1mL of blood was collected using a 27G needle and transferred into tubes with 100µl of EDTA (0.5M, pH 8) solution. The blood was transferred into 5mL RIA/FACS polystyrene tubes with the addition of 1,5 x of total volume Tyrodes buffer (134mM NaCl, 2.9mM KCl, 0.34mM Na2HPO4.2H20, 12mM NaHCO3, 20mM HEPES, 1mM MgCl2.6H20 and 5mM D-Glucose anhydrous). Tubes were centrifuged (100g, 20min) and the platelet rich-plasma layer was collected, in the presence of 0.5u/ml of apyrase (A6410, Merck Sigma- Aldrich) and 50ng/ml of PGE1 (P5515, Merck Sigma-Aldrich). After another centrifugation (1000g, 10min) the pellet was resuspended in NSPC or OPC culture medium. For labelling with the lipophilic dye PKH26 (PKH26GL, Merck Sigma-Aldrich), platelets were incubated (1mM in 100% ethanol, 4min at room temperature), according to the manufacturer's protocol and in the presence of PEG1.

To assess the activation of platelets, 50µL of platelet suspension were incubated with allophycocyanin (APC)-labeled anti-mouse CD41 (1:100, 133913, BioLegend) and fluorescein isothiocyanate (FITC) rat anti-mouse CD62P (1:100, 553744, BD Pharmingen) or the FITC-rat IgG1λ isotype control (1:100, 553995, BD Pharmingen), for 20min. Samples were analyzed using the BD Accuri TM C6 Plus (BD Biosciences) in a 35 µl/min flowrate.

The MTT assay was used to assess the activity of mitochondria in platelets, as an indicator of their activation that leads to their exhaustion. We followed the protocol provided by the provider (CyQUANT MTT, Thermosfisher), with 5mg/ml of the yellow tetrazolium salt MTT (3-(4,5-dimethylthiazol-2-yl)-2,5-diphenyltetrazolium bromide) added to 96 well plates in which platelets had been plated on PDL. The incubation lasted 4h and was followed by the removal of the medium and the addition of DMSO. Optical absorbance was measured at 545nm, using a microplate reader.

Isolation and culture of NSPCs and of OPCs; co-cultures

For mouse NSPC isolation, bilateral dissections of the brain's subependymal zones were performed under the stereoscope using anatomical landmarks, as done previously[2] and tissue was dissociated using accutase (25-

058-CI, Corning). Cells were plated in T25 flasks in NSPC proliferation medium that is composed of high glucose DMEM (11995065, ThermosFisher Scientific), FGF2 (00-18B, PEPROTECH) and EGF (315-09, PEPROTECH) at 20ng/ml, 2% B27 (17504044, ThermosFisher Scientific) and 1% N2 (17502048, ThermoFisher Scientific) supplements and 1% penicillin/streptomycin (15070063, ThermoFisher Scientific). Cells were allowed to grow as 3D free-floating aggregates called neurospheres that were passaged, every 5 to 7 days, between 5 and 10 times.

Primary OPC cultures were obtained from postnatal day 2 mouse cortices, as previously described [**67**]. Briefly, cortices were diced into small pieces, after the removal of meninges, and were mechanically dissociated. Cells were plated onto poly-D-lysine (100 mg/ml, Merck Sigma– Aldrich, A-003-E) coated T75 culture flasks and cultured in DMEM (Glutamax™, 61965-026, 14.5g/L d-Glucose, Pyruvate, ThermoFisher Scientific), supplemented with 10% FBS (F1051, Merck) and 1% pen/strep (P4458, Merck). The culture medium was replenished twice a week, until mixed glial cultures became confluent (after 10–12 days). At this point the microglial cells were removed using an orbital shaker at 200 rpm for 1 h at 37°C and OPC population was separated from the underlying astrocytic cell layer by vigorous shaking (16 hr at 240 rpm, 37°C). OPCs were then washed and seeded at an initial density of 35,000 cells per well in 48-well plates containing 9mm glass coverslips. All plates were previously coated overnight with poly-D-lysine. OPCs were cultured in DMEM, supplemented with 1% N2, 1 µM D-biotin (B4501, Merck Sigma-Aldrich), 1% BSA fatty acid-free (Merck Sigma-Aldrich), 5 µg/ml Nacetylcycteine (A8199, Merck Sigma-Aldrich), 1% penicillin/streptomycin, PDGF-AA (10ng/ml , 100-13A, PEPROTECH) and hFGF-2 (10 ng/ml, 10018B, PEPROTECH) to allow the proliferation of OPCs. After 3 days medium was changed to differentiation medium containing 40ng/ml T3 (T6397, Merck Sigma-Aldrich), instead of PDGF-AA and hFGF-2, to allow the immediate differentiation of OPCs toward mature OLs. The day of cell plating is considered as DIV0.

Co-cultures were performed in multi-well plates, with cells grown on glass coverslips, coated with PDL. NSPCs or OPCs were dissociated and plated the day before the addition of platelets. A range of cell:platelet ratios were used (1:40, 1:75, 1:400, 1:1000, 1:1200, 1:3000) and the cultures were left for 3 (NSPCs) or 4 (OPCs) days, without addition or change of medium.

Supplementary Material

Transient thrombocytopenia

For the chemical depletion of platelets, mice were injected twice, i.p. and under inhaled anesthesia (2.5% Isoflurane), with anti-mouse CDCD42 antibody (0.6µg/g of body weight, diluted in sterile PBS; #RR300, Emfret Analytics, GMBH) or vehicle (sterile PBS). The first injection was performed three days after the induction of demyelination and it was repeated after 48h (at 3dpl and 5dpl). Numbers of circulating platelets were monitored before the day of antibody injection, and subsequently at 1-, 2- and 4-days post-injection. Approximately 80µL of blood were collected from a cut at the tip of the tail, into Microvettes (SARSTEDT CB 300 KK2EE, #16.444) and platelets were counted with an automated veterinary blood analyser.

RNA-seq

Whole transcriptome sequencing analysis was performed in postnatal mouse brain Neural Stem Cells (NSCs) cultured on PDL-coated wells of 6-well plates. Three independent cell cultures were performed (sample 1 derived form a 2 month-old male mouse; sample 2 derived from a 3 month-old female mouse; sample 3 derived from a 2.5 month-old male mouse) with 300000 cells plated, immediately after dissociation of passage 5 neurospheres, in wells of 6-well plates and kept overnight in proliferation medium to achieve good adherence. The next day one well (per sample) was kept in proliferation conditions, in a second well the medium was changed to differentiation medium and in the third 22500000 platelets (ratio 1:75) were added in differentiation medium. The cultures were kept for three days, without any media changes or additions. Total RNA extraction was performed with the NucleoSpin RNA Plus kit (MACHEREY-NAGEL, 740984), according to the manufacturer's instructions. RNA isolation from cells cultured in the other two conditions was performed with the NucleoSpin RNA Plus XS kit (MACHEREY-NAGEL, 740990), according to the manufacturer's instructions. The ribosomal RNA was depleted using the RiboMinusTM Eukaryote Kit v2 (ThermoFisher Scientific Inc., A15020) and quantification of the RNAs was performed using the Qubit RNA HS (High Sensitivity) Assay Kit (ThermoFisher Scientific Inc., Q32852) with the Qubit Fluorometer. 100 ng of rRNA-depleted total RNA were used for the preparation of the cDNA libraries using the Ion Total RNA-Seq v2 Kit (ThermoFisher Scientific Inc., 4475936) according to the manufacturer's instructions, while a unique barcode was added in each fragmented library using the Ion Xpress™ RNA-Seq Barcode 1-16 Kit (ThermoFisher Scientific Inc., 4471250). The quantitation of the libraries was performerd with the Qubit 1x dsDNA HS Assay Kit (ThermoFisher Scientific Inc., Q33231) and their quality was assessed using the Agilent High Sensitivity DNA Kit (Agilent Technologies, 5067-4626) on the 2100 Bioanalyzer system. Template preparation and Ion 540™ chip (ThermoFisher Scientific Inc., A27766) loading were performed on the Ion Chef system with the Ion 540™ Chef

kit (ThermoFisher Scientific Inc., A30011). The single-end sequencing was carried out on the Ion GeneStudio S5 system.

Analysis of RNA-seq data

The analysis of the sequencing data was performed on the Galaxy web platform through the usegalaxy.org public server (Afgan et al 2018). Reads were mapped to mm10 with the STAR aligner (v2.7.8a) with default parameters except the following (--sjdbOverhang 75 --outSAMmapqUnique 255 --chimSegmentMin 18 --chimScoreMin 12 --outFilterType BySJout) (Dobin et al., 2013). The unmapped reads were aligned to mm10 using bowtie2 (v2.4.5) using the --very-sensitive-local preset (Langmead and Salzberg, 2012). The generated BAM files were then merged using picard MergeSamFiles (v2.18.2.1) followed by filtering out the unmapped reads with the samtools view (v1.15.1) (Li et al., 2009)(Broad Institute, 2009). Gene expression was counted with featureCounts (v2.0.1) and the differential expression analysis was performed with limma-voom (v3.50.1), with genes with CPM>0.5 in at least one sample only considered (Liao et al., 2014; Liu et al., 2015). All raw and processed sequencing data are available at the GEO Repository under the Accession Number GSE256325. Further functional enrichment analysis of the differentially expressed genes was performed on the PANTHER platform (Thomas et al., 2022).

RT-PCR

Cell lysis and RNA isolation was performed according to the manufacturer protocols using the Kit NucleoSpin RNA Plus kit (MACHEREYMACHEREY-NAGELNAGEL, 740984) for NSPCs in proliferation conditions and with the NucleoSpin RNA Plus XS kit (MACHEREYMACHEREY-NAGELNAGEL, 740990) for NSPCs in (the less dense) differentiation conditions. cDNA was generated using the High-Capacity cDNA Reverse Transcription Kit (Applied Biosystems, cat. num. 4374966) and 200ng of RNA. Real time PCR was performed using the KAPA SYBR FAST Universal qPCR Kit (KAPA BiosystemsBiosystems, KKKK4600) with actin-b and GAPDH acting as housekeeping, reference, genes. The primers used were the following:

mKi67 forward	CCTTTGCTGTCCCCGAAGA
mKi67 reverse	GGCTTCTCATCTGTTGCTTCCT
mSox10 forward	AGATGGGAACCCAGAGCAC
mSox10 reverse	CTCTGTCTTTGGGGTGGTTG

Supplementary Material

β-actin forward	AGCTCCTTCGTTGCCGGTCC
β-actin reverse	TCGGTGAGCAGCACAGGGTG
GAPDH forward	GAGAAACCTGCCAAGTA
GAPDH reverse	AGACAACCTGGTCCTCA

Immunocytochemistry

Cells were fixed using pre-heated PFA (Merck Sigma-Aldrich, 16005) in a final 2% concentration for 15 min at room temperature and were immunostained using standard precedures[2]. Washing with 1x PBS was followed by incubation of the cells in blocking solution of 1% BSA (PanReac AppliChem, A1391), 0.1% TritionX-100 in 1x PBS for 30 min at RT. Afterwards, cells were incubated with primary antibodies in blocking solution for 1.5 h at RT. 10 minutes washing with 1x PBS was followed by labelling of the cells with the appropriate secondary fluorescent antibodies (from Biotium and ThermoFisher Scientific/ Molecular Probes) and DAPI (1:1500, Invitrogen, MP01306) in blocking solution for 45 min at RT. Finally, cells were washed in PBS for 10 minutes and coverslips were mounted with mounting medium containing Mowiol® 4-88 Reagent (Millipore, 475904).

Primary antibodies used were:

Mouse anti-adenomatous polyposis coli clone CC1 (APC/CC-1) (1:100, Merck Millipore, OP80)

Rat, anti-MBP (1:200, Serotec, MCA409S)

Goat, anti-Sox2 (1:200, R&D systems, AF2018

Rabbit, anti-Dcx (1:500, Abcam, ab18723)

Rabbit, anti-Olig2 (1:200, Merck Millipore, AB9610)

Rabbit, anti-Ki67 (1:500, Merck Sigma-Aldrich, AB9260)

Rat, anti-CD41 (1:200, BD Pharmingen, 553848)

Chicken, anti-Nestin (1:500, Abcam, 130417)

Mouse, anti-PCNA (1:500, Abcam, ab29)

Supplementary Material

Immunohistochemistry

Mice were transcardially perfused with ice-cold PBS (20ml) followed by ice-cold 4% PFA (50ml, in PBS; Merck Sigma-Aldrich, 16005). Forebrains were dissected, post-fixed (4% PFA, 4°C, overnight), cryoprotected (30% sucrose, 0.1% NaN3 in PBS) and frozen at -80°C. They were sectioned (Leica) in 14-µm-thick coronal sections. Immunofluorescence was performed with standard protocols[2], using the primary antibodies listed below and the appropriate secondary antibodies (from Biotium and ThermoFisher Scientific/ Molecular Probes) and with nuclear counterstaining using DAPI (1:1500, Invitrogen, MP01306):

Rabbit, anti-PLP (1:1000, Abcam, ab28486)

Rat, anti-PDGFRa (1:100, Merck Millipore, CBL1366, RRID:AB11211998)

Rabbit, anti Laminin (1:1000, Abcam, ab11575)

Rat, anti-CD41 (1:200, BD Pharmingen, 553848)

Mouse, anti-TH (1:1000, Cell Signalling, 3873)

Mouse, anti-PCNA (1:500, Abcam, ab29)

Goat, anti-Sox2 (1:200, R&D systems, AF2018

Rabbit, anti-Olig2, (1:200, Merck Millipore, AB9610)

Rabbit, anti-Dcx (1:500, Abcam, ab18723)

Rabbit, anti-Ki67, (1:500, Merck Sigma-Aldrich, AB9260)

Goat, anti-GFAP (1:500, Abcam, ab53554)

Goat, anti-Iba1 (1:500, Abcam, ab5076)

Rabbit, anti PH3 (1:500, Abcam, ab80612)

Supplementary References

1. Im, J. & Muschel, R. Protocol for Murine/Mouse Platelets Isolation and Their Reintroduction in vivo. *BIO-PROTOCOL* **7**, (2017).
2. Kazanis, I. *et al.* Subependymal Zone-Derived Oligodendroblasts Respond to Focal Demyelination but Fail to Generate Myelin in Young and Aged Mice. *Stem Cell Reports* **8**, 685–700 (2017).
