# Supplementary material for: Platelets regulate neural and oligodendroglial progenitors when infiltrating the brain parenchyma
Source: Commun Biol. 2025 Nov 24;8:1640. doi: 10.1038/s42003-025-09028-1 (PMC12644571; doi:10.1038/s42003-025-09028-1)
Supplement: Supplementary file 3 — Description of Additional Supplementary files [file 42003_2025_9028_MOESM3_ESM.pdf]

Description of Additional Supplementary files

File name: Supplementary Data 1

Description: Numerical data for graphs reporting on in vitro work (Figures 1, 3 & Sup Figure 1, Sup Fig 2, Sup Fig 5)

File name: Supplementary Data 2

Description: Numerical data for graphs reporting in in vivo work (Figures 3, 4, 5)

File name: Supplementary Video 1

Description: 3D reconstruction of a blood vessel, identified by the expression of laminin (in red) with platelets identified by expression of CD41 (in green), within the SEZ niche of a wild-type mouse after the induction of a demyelinating lesion in the adjacent corpus callosum. Cell nuclei are counterstained with DAPI (in blue).
