# Supplementary material for: Platelets regulate neural and oligodendroglial progenitors when infiltrating the brain parenchyma
Source: Commun Biol. 2025 Nov 24;8:1640. doi: 10.1038/s42003-025-09028-1 (PMC12644571; doi:10.1038/s42003-025-09028-1)
Supplement: Supplementary file 7 — Reporting Summary [file 42003_2025_9028_MOESM7_ESM.pdf]

nature portfolio

Corresponding author(s): Ilias Kazanis

Last updated by author(s): Oct 4, 2025

Reporting Summary

Nature Portfolio wishes to improve the reproducibility of the work that we publish. This form provides structure for consistency and transparency in reporting. For further information on Nature Portfolio policies, see our Editorial Policies and the Editorial Policy Checklist.

Statistics

For all statistical analyses, confirm that the following items are present in the figure legend, table legend, main text, or Methods section.

n/a	Confirmed	
☐	☒	The exact sample size (*n*) for each experimental group/condition, given as a discrete number and unit of measurement
☐	☒	A statement on whether measurements were taken from distinct samples or whether the same sample was measured repeatedly
☐	☒	The statistical test(s) used AND whether they are one- or two-sided<br>*Only common tests should be described solely by name; describe more complex techniques in the Methods section.*
☐	☒	A description of all covariates tested
☒	☐	A description of any assumptions or corrections, such as tests of normality and adjustment for multiple comparisons
☐	☒	A full description of the statistical parameters including central tendency (e.g. means) or other basic estimates (e.g. regression coefficient) AND variation (e.g. standard deviation) or associated estimates of uncertainty (e.g. confidence intervals)
☐	☒	For null hypothesis testing, the test statistic (e.g. *F*, *t*, *r*) with confidence intervals, effect sizes, degrees of freedom and *P* value noted<br>*Give P values as exact values whenever suitable.*
☒	☐	For Bayesian analysis, information on the choice of priors and Markov chain Monte Carlo settings
☒	☐	For hierarchical and complex designs, identification of the appropriate level for tests and full reporting of outcomes
☒	☐	Estimates of effect sizes (e.g. Cohen's *d*, Pearson's *r*), indicating how they were calculated

Our web collection on statistics for biologists contains articles on many of the points above.

Software and code

Policy information about availability of computer code

Data collection — *Provide a description of all commercial, open source and custom code used to collect the data in this study, specifying the version used OR state that no software was used.*

Data analysis — *Provide a description of all commercial, open source and custom code used to analyse the data in this study, specifying the version used OR state that no software was used.*

For manuscripts utilizing custom algorithms or software that are central to the research but not yet described in published literature, software must be made available to editors and reviewers. We strongly encourage code deposition in a community repository (e.g. GitHub). See the Nature Portfolio guidelines for submitting code & software for further information.

Data

Policy information about availability of data

All manuscripts must include a data availability statement. This statement should provide the following information, where applicable:

- Accession codes, unique identifiers, or web links for publicly available datasets
- A description of any restrictions on data availability
- For clinical datasets or third party data, please ensure that the statement adheres to our policy

All the raw data, supporting the findings of this study are not openly available due to reasons of sensitivity, but can be made available upon reasonable request to the corresponding author. RNA-sequencing raw data are available from the Gene Expression Omnibus under accession code GSE256325.

Research involving human participants, their data, or biological material

Policy information about studies with human participants or human data. See also policy information about sex, gender (identity/presentation), and sexual orientation and race, ethnicity and racism.

Reporting on sex and gender	*Use the terms sex (biological attribute) and gender (shaped by social and cultural circumstances) carefully in order to avoid confusing both terms. Indicate if findings apply to only one sex or gender; describe whether sex and gender were considered in study design; whether sex and/or gender was determined based on self-reporting or assigned and methods used. Provide in the source data disaggregated sex and gender data, where this information has been collected, and if consent has been obtained for sharing of individual-level data; provide overall numbers in this Reporting Summary. Please state if this information has not been collected. Report sex- and gender-based analyses where performed, justify reasons for lack of sex- and gender-based analysis.*
Reporting on race, ethnicity, or other socially relevant groupings	*Please specify the socially constructed or socially relevant categorization variable(s) used in your manuscript and explain why they were used. Please note that such variables should not be used as proxies for other socially constructed/relevant variables (for example, race or ethnicity should not be used as a proxy for socioeconomic status). Provide clear definitions of the relevant terms used, how they were provided (by the participants/respondents, the researchers, or third parties), and the method(s) used to classify people into the different categories (e.g. self-report, census or administrative data, social media data, etc.) Please provide details about how you controlled for confounding variables in your analyses.*
Population characteristics	*Describe the covariate-relevant population characteristics of the human research participants (e.g. age, genotypic information, past and current diagnosis and treatment categories). If you filled out the behavioural & social sciences study design questions and have nothing to add here, write "See above."*
Recruitment	*Describe how participants were recruited. Outline any potential self-selection bias or other biases that may be present and how these are likely to impact results.*
Ethics oversight	*Identify the organization(s) that approved the study protocol.*

Note that full information on the approval of the study protocol must also be provided in the manuscript.

Field-specific reporting

Please select the one below that is the best fit for your research. If you are not sure, read the appropriate sections before making your selection.

☒ Life sciences ☐ Behavioural & social sciences ☐ Ecological, evolutionary & environmental sciences

For a reference copy of the document with all sections, see nature.com/documents/nr-reporting-summary-flat.pdf

Life sciences study design

All studies must disclose on these points even when the disclosure is negative.

Sample size	When applying for ethical apporval, we used power calculations to estimate the number of animals that we would need to include in this study.<br>We compared three time-points after demyelination and for each variable (e.g. total cell density, percentage of of proliferating cels) we had two values per animal (ipsilateral and contralateral to demyelination, as internal control). We included three experimental groups (wild-type, chemical depletion of platelets, Nbeal2 knockout). Based on previous experimental work, we expected that differences in averages at the level of 20% (and above) would generate statistically significant results (based on Kazanis et al., 20017; McClenahan et al., 2023). The significance level was et at $p<0.05$ and the power at $\pi=0.85$ and we used multiple ANOVA (MANOVA, repeated measures, within-between interaction). Following the approximation method (F-transformation) (Pillai and Mijares, 1959) and the O'Brien and Shieh (1999) algorithm, the total number of mice was calculated as n=25, i.e. 3-5 mice per time-point.
Data exclusions	No data were excluded
Replication	Experiments were analysed blind and were repeated by different researchers
Randomization	All mice (for the isolation of neural stem cells or platelets, as well as for the chemical depletion of platelets) were randomly selected from the available colonies. The experiments on the Nbeal2 colony were performed after establishing the genotype (wild-type, or knockout animals) using all the available knockout mice and randomly selecting wild-type mice.
Blinding	Investigators were blinded during data analyis (cell counting). Where this was not possible (MTT assay, intracerebroventircular injection of platelets) the counts were performed by two independent investigators. Many of the experiments and analyses (especially the invitro, co-culture, assays) were perfromed by different investigators at different labs and times and te results include all these data.

Reporting for specific materials, systems and methods

We require information from authors about some types of materials, experimental systems and methods used in many studies. Here, indicate whether each material, system or method listed is relevant to your study. If you are not sure if a list item applies to your research, read the appropriate section before selecting a response.

Materials & experimental systems

n/a	Involved in the study	
☐	☒	Antibodies
☒	☐	Eukaryotic cell lines
☒	☐	Palaeontology and archaeology
☐	☒	Animals and other organisms
☒	☐	Clinical data
☒	☐	Dual use research of concern
☒	☐	Plants

Methods

n/a	Involved in the study	
☒	☐	ChIP-seq
☐	☒	Flow cytometry
☒	☐	MRI-based neuroimaging

Antibodies

Antibodies used	Mouse anti-adenomatous polyposis coli clone CC1 (APC/CC-1) (1:100, Merck Millipore, OP80)<br>Rat, anti-MBP (1:200, Serotec, MCA409S)<br>Goat, anti-Sox2 (1:200, R&D systems, AF2018<br>Rabbit, anti-Dcx (1:500, Abcam, ab18723)<br>Rabbit, anti-Olig2 (1:200, Merck Millipore, AB9610)<br>Rabbit, anti-Ki67 (1:500, Merck Sigma-Aldrich, AB9260)<br>Rat, anti-CD41 (1:200, BD Pharmingen, 553848)<br>Chicken, anti-Nestin (1:500, Abcam, 130417)<br>Mouse, anti-PCNA (1:500, Abcam, ab29)<br>Rabbit, anti-PLP (1:1000, Abcam, ab28486)<br>Rat, anti-PDGFRa (1:100, Merck Millipore, CBL1366, RRID:AB11211998)<br>Rabbit, anti Laminin (1:1000, Abcam, ab11575)<br>Mouse, anti-TH (1:1000, Cell Signalling, 3873)<br>Goat, anti-Iba1 (1:500, Abcam, ab5076)<br>Rabbit, anti PH3 (1:500, Abcam, ab80612)
Validation	Experimental data using all the antibodies included in this manuscript have been reported in multiple previous publications from our groups:<br>CC1, MBP, PLP, PDGFRalpha (https://doi.org/10.1002/jnr.24809, https://doi.org/10.1111/ejn.15121, https://doi.org/10.7554/eLife.91757.3, https://doi.org/10.1016/j.stemcr.2017.01.007)<br>Sox2, Dcx, Olig2, Ki67, CD41, Nestin, PCNA, Laminin, Iba1, PH3 (https://doi.org/10.1016/j.stemcr.2017.01.007, https://doi.org/10.1016/j.expneurol.2015.03.018, https://doi.org/10.1016/j.expneurol.2013.06.025,)<br>TH (https://doi.org/10.1186/s13287-021-02398-3, https://doi.org/10.4103/1673-5374.385314)

Animals and other research organisms

Policy information about studies involving animals; ARRIVE guidelines recommended for reporting animal research, and Sex and Gender in Research

Laboratory animals	Male and female mice of the Bl6CBAC or of the 129sv background. Male mice of the Nbeal2 transgenic line (https://doi.org/10.1182/blood-2014-04-566760). Male Sprague Dawley rats.
Wild animals	The study did not involve wild animals.
Reporting on sex	For all in vitro experiments male and female mice were used.<br>For the in vivo experiments involving the Nbeal2 transgenic mice colony, only male mice were used (as declared in materials and methods). This was due to colony-maintenance issues (knockout females were necessary for breeding pairs).<br>Rat brain tissue samples of the MCAO experiment were from male mice (as desctribed in materials and methods).
Field-collected samples	The study did not involve samples from animals collected in the field.
Ethics oversight	Animal breeding, maintenance and handling was performed in accordance with the European Communities Council Directive Guidelines (86/609/EEC) for the care and use of Laboratory animals as implemented in Greece by the Presidential Decree 56/2013 and approved and scrutinized by the local Prefectural Animal Care and Use Committee in Patras (Protocol number: 118188/432/21-05-2020) and in Crete (Protocol number: 106357/31-05-2021).

Note that full information on the approval of the study protocol must also be provided in the manuscript.

Plants

Seed stocks

Report on the source of all seed stocks or other plant material used. If applicable, state the seed stock centre and catalogue number. If plant specimens were collected from the field, describe the collection location, date and sampling procedures.

Novel plant genotypes

Describe the methods by which all novel plant genotypes were produced. This includes those generated by transgenic approaches, gene editing, chemical/radiation-based mutagenesis and hybridization. For transgenic lines, describe the transformation method, the number of independent lines analyzed and the generation upon which experiments were performed. For gene-edited lines, describe the editor used, the endogenous sequence targeted for editing, the targeting guide RNA sequence (if applicable) and how the editor was applied.

Authentication

Describe any authentication procedures for each seed stock used or novel genotype generated. Describe any experiments used to assess the effect of a mutation and, where applicable, how potential secondary effects (e.g. second site T-DNA insertions, mosiacism, off-target gene editing) were examined.

Flow Cytometry

Plots

Confirm that:

☒ The axis labels state the marker and fluorochrome used (e.g. CD4-FITC).

☒ The axis scales are clearly visible. Include numbers along axes only for bottom left plot of group (a 'group' is an analysis of identical markers).

☒ All plots are contour plots with outliers or pseudocolor plots.

☒ A numerical value for number of cells or percentage (with statistics) is provided.

Methodology

Sample preparation

In order to investigate levels of activation of washed isolated platelets, we performed flow cytometry (FC) analysis. Therefore, 10µl from each platelet sample (resuspended in neural stem cell medium) were added in 190µl of FC buffer (2% BSA, 2 mM EDTA in PBS Dulbecco) in each FC tube and were incubated with the following antibodies: a) allophycocyanin (APC)-labeled anti-mouse CD41 (1:100, 133913, BioLegend) and b) fluorescein isothiocyanate (FITC) rat anti-mouse CD62P (1:100, 553744, BD Pharmingen), for 20min in the dark. Furthermore, a control isotype staining for CD62P was also performed: 10µl of platelet sample were added in 190µl of FC buffer and were incubated with the APC-labeled anti-mouse CD41 and the FITCrat IgG1λ isotype control (1:100, 553995, BD Pharmingen) antibodies. The reaction of the staining was stopped with the addition of FC buffer (1ml) and samples were analysed with the Flow Cytometer: BD Accuri TM C6 Plus (BD Biosciences).

Instrument

Flow Cytometer: BD Accuri TM C6 Plus (BD Biosciences)

Software

BD Accuri C6 Software

Cell population abundance

Platelets (the cell population under investigation) constituted approximately 99% of events.

Gating strategy

Based on Forward and Side scattering patterns of platelets, as well as by the CD41+ staining, we defined the population of platelets. Samples were analyzed at a moderate flow rate (Flow rate: 35 µl/min), using a threshold for forward scattering at 25,000 in order to include platelets while excluding small debris. The population of activated platelets was defined as CD41 +CD62P+ platelets (CD41+ platelets were gated for the expression of the platelet activation marker CD62P+) and the % percentage of activated platelets was evaluated, subtracting the %percentage of positive platelets identified from the isotype control staining.

☒ Tick this box to confirm that a figure exemplifying the gating strategy is provided in the Supplementary Information.
